# 3D Structural View of Pathogen Recognition by Mammalian Lectin Receptors

**DOI:** 10.3389/fmolb.2021.670780

**Published:** 2021-05-25

**Authors:** Noriyoshi Manabe, Yoshiki Yamaguchi

**Affiliations:** Institute of Molecular Biomembrane and Glycobiology, Division of Structural Glycobiology, Tohoku Medical and Pharmaceutical University, Sendai, Japan

**Keywords:** mammalian lectin receptor, microbe glycan, 3D structure, interaction, non-self

## Abstract

Humans and other mammals resist exogenous pathogens by recognizing them as non-self. How do they do this? The answer lies in the recognition by mammalian lectin receptors of glycans usually found on the surface of pathogens and whose chemical structure is species-specific. Some glycan components, such as galactofuranose, only occur in microbes, and is the principal means by which mammalian lectin receptors recognize non-self. Several lectins may function together as pattern recognition receptors to survey the infecting pathogen before the adaptive immune system is invoked. Most lectins have primary and secondary monosaccharide-binding sites which together determine the specificity of a receptor toward microbial glycans. There may also be a hydrophobic groove alongside the sugar binding sites that increases specificity. Another elaboration is through oligomerization of lectin domains with defined spacing and arrangement that creates high-affinity binding towards multiply-presented glycans on microbes. Microbe-specific polysaccharides may arise through unique sugar linkages. Specificity can come from mammalian receptors possessing a shallow binding site and binding only internal disaccharide units, as in the recognition of mannan by Dectin-2. The accumulation of 3D structural information on lectins receptors has allowed the recognition modes of microbe glycans to be classified into several groupings. This review is an introduction to our current knowledge on the mechanisms of pathogen recognition by representative mammalian lectin receptors.

## Introduction

**FIGURE 1:**
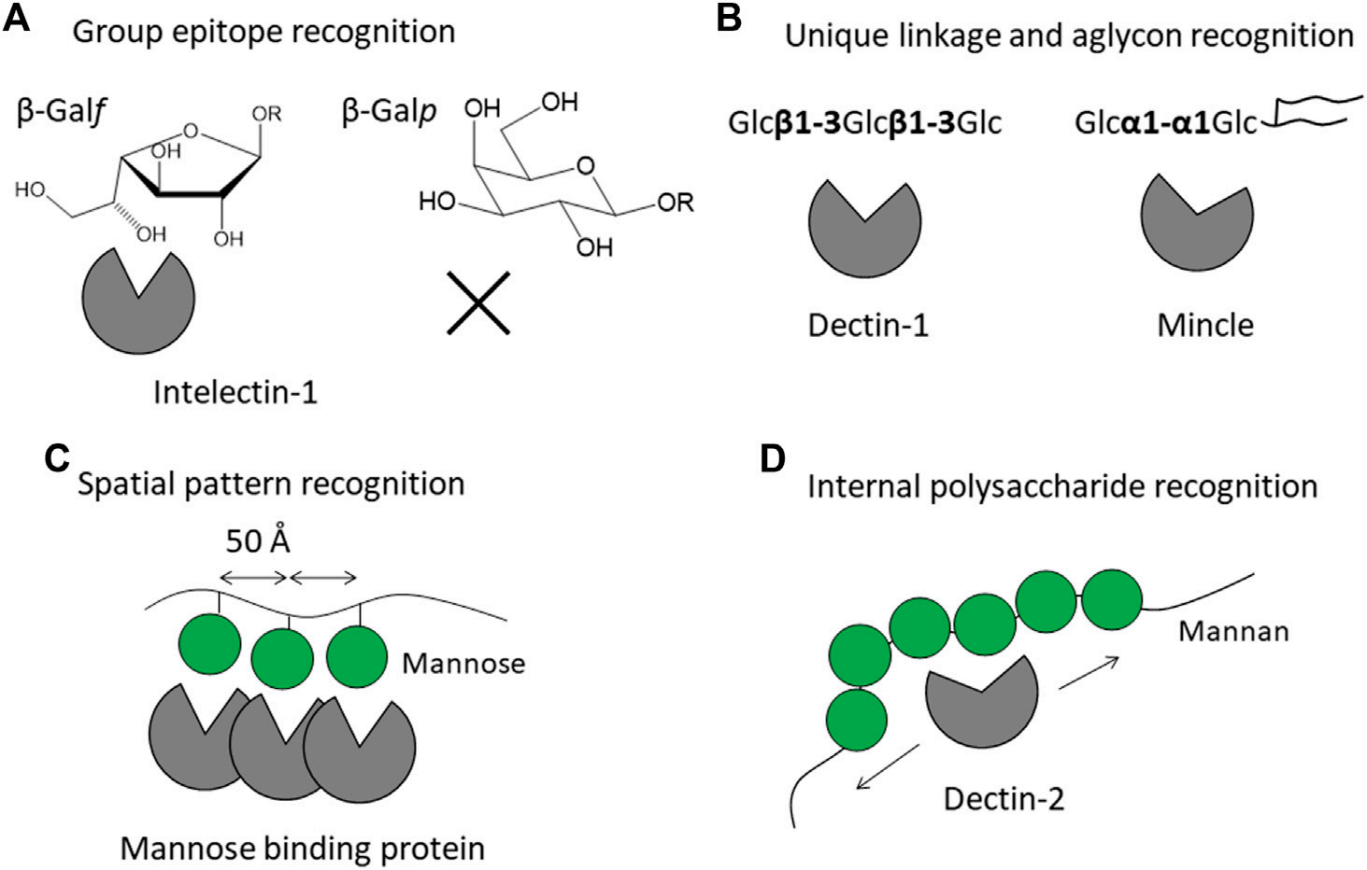
Strategies of mammalian lectin receptors for binding to microbe glycans (non-self) but not to endogenous glycans (self). Recognition modes are classified into four patterns: **(A)** recognition of specific group epitope as exemplified by galactofuranose recognition by intelectin-1, **(B)** recognition of oligosaccharide with unique linkage and aglycon as exemplified by β-glucan recognition by Dectin-1 and trehalose recognition by Mincle, **(C)** pattern recognition of special arrangement of sugar residues as exemplified by trimeric mannose-binding protein, and **(D)** internal recognition of polysaccharide as exemplified by Dectin-2 binding to mannan. Mannose is shown as a green filled circle, adopted from Symbol Nomenclature for Glycans (SNFG) (Neelamegham et al., 2019).

Glycans are covalently linked to proteins or lipids and are mostly expressed on the cell surface. The glycan structures are highly diverse and species-specific, in contrast to other biomolecules such as proteins and nucleic acids. Pathogens often express glycans which do not occur in humans and other mammals. The mammalian immune system has the ability to recognize pathogen-specific glycans, and in doing so an immune response is triggered. Lectin receptors are on the front line and play a direct role in detecting the glycans. Lectin-glycan interaction is generally weak and hence the specificity is low. One strategy to overcome the inherent weak affinity is to form a lectin oligomer gaining its increased affinity toward multiply presented glycans on microbes. How then do mammalian lectin receptors distinguish such exogenous glycans from the abundant endogenous glycans? The answer is provided by 3D structures and interaction modes of the mammalian lectin receptors. 3D structures of lectin receptors are deposited in the Protein Data Bank (PDB), and we could see how the glycan is recognized by lectin receptors. Knowledge on 3D structures of lectins is accumulating and summarized with curated information in UniLectin3D (Bonnardel et al., 2019). Here we focus on mammalian lectin receptors whose atomic 3D structures and binding modes toward the glycans of pathogens are available. This review is not a comprehensive list and analysis of all mammalian lectin receptors, the aim is rather to categorize the recognition modes of the glycans of pathogens, to gain an understanding of the structural basis of self/non-self-discrimination. Lectins are often categorized by their structural fold and monosaccharide-binding specificity. We here attempt to classify the recognition modes of microbe glycan into four modes: i) recognition of specific group epitope, ii) recognition of oligosaccharide with unique linkage and aglycon, iii) pattern recognition of special arrangements of sugar residues, and iv) internal recognition of polysaccharide (Figure 1). This classification is a starting point for discussion and will be revised according to new findings and inputs. Hereafter these recognition modes are explained by means of 3D structures of representative lectin receptors (Table 1).

**TABLE 1:** List of mammalian lectin receptors described in this review.

recognition Mode	lectin	Species	PDB ID^a^	UniLectin3D ID^b^	ligand	Reference
Group epitope recognition	Intelectin-1	Human	4WMY	Q8WWA0	Allyl-β-D-Gal*f*	Wesener et al. (2015)
	Intelectin-1	Human	6USC	Q8WWA0	α-KO	McMahon et al. (2020)
	SP-D	Human	2RIB	P35247	l-glycero-d-manno-heptose	Wang et al. (2008)
Unique linkage /aglycon recognition	Dectin-1	Mouse	2BPE	Q6QLQ4	–	Brown et al. (2007)
	Mincle	Cow	4ZRV	E1BHM0	Trehalose	Feinberg et al. (2016)
	DCAR	Mouse	6LFJ	–	Phosphoglycolipids	Omahdi et al. (2020)
	ZG16p	Human	3VY7	O60844	αMan-O-Ser	Kanagawa et al. (2014)
Spatial pattern recognition	MBP-A trimer	Rat	1KWU	P19999	αMan-O-Me	Ng et al. (2002)
	Langerin trimer	Human	3KQG	Q9UJ71	–	Feinberg et al. (2010)
	Langerin monomer	Human	3P5D	Q9UJ71	Manα1-3Man	Feinberg et al. (2011)
Internal polysaccharide recognition	Dectin-2	Human	5VYB	Q6EIG7	Manα1-2Manα1-3(Manα1-2Manα1-6)Man	Feinberg et al. (2017)

a PDB: https://www.rcsb.org/

b Unilectin3D:https://unilectin.eu/unilectin3D/

## Recognition of Specific Group Epitope

**FIGURE 2:**
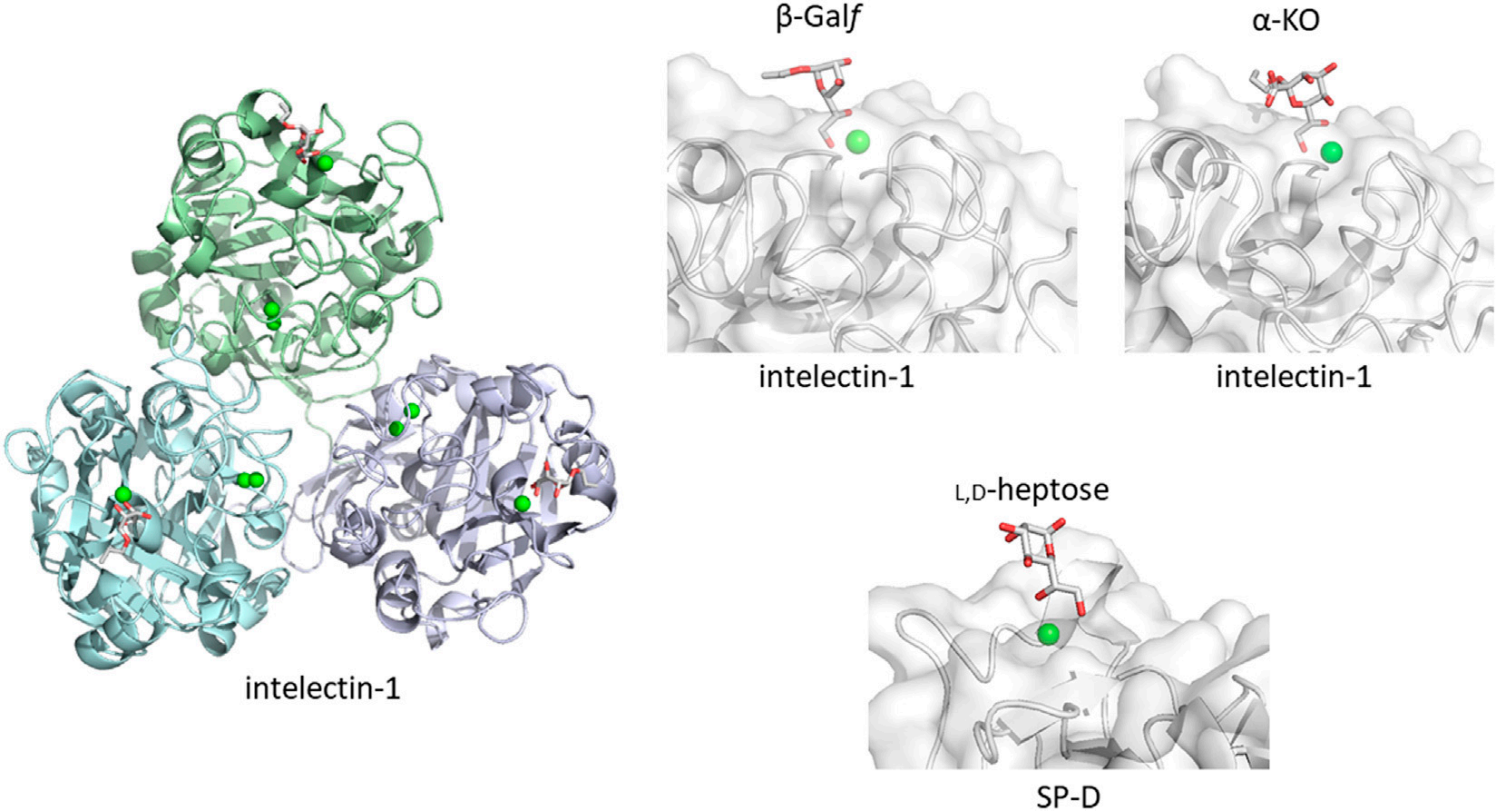
Galactofuranose and α-KO recognition by human intelectin-1 **(A)**. Overall structure of trimeric intelectin-1 in complex with allyl-β-D-galactofuranose (β-Gal*f*) (**(B)**, PDB ID: 4WMY) and close-up view of ligand-binding site with β-Gal*f* or α-KO (**(C)**, PDB ID: 6USC). 3D structure of SP-D with L,D-heptose is shown for comparison (**(D)**, PDB ID: 2RIB). Calcium ion is shown as a green sphere and the ligand in stick representation.

How does the mammalian immune system sense microbe glycans as non-self? One possible simple and straightforward strategy is to detect microbe-specific sugar residues not found in mammals. An example is galactofuranose recognition by intelectin-1. Galactofuranose is a five-membered ring monosaccharide found in microbes but not in humans. Targeting galactofuranose as a non-self component is therefore a reasonable strategy to detect an invader. Human intelectin-1 shows unique specificity towards furanose residues, including ribofuranose and β-galactofuranose-containing disaccharide (Tsuji et al., 2001; Tsuji et al., 2007). In a detailed glycan microarray analysis, human intelectin-1 binds not only to β-linked D-galactofuranose but to other microbe-specific glycan components: e.g., α-KO (D-glycero-D-talo-oct-2-ulosonic acid) and α-KDO (3-deoxy-D-manno-oct-2-ulosonic acid) (Wesener et al., 2015). Human intelectin-1 does not bind mammalian glycans. We have crystal structures of human intelectin-1 bound to allyl-β-D-galactofuranose or allyl-α-KO at the resolution of 1.6 Å (Wesener et al., 2015; McMahon et al., 2020) (Figure 2). The lectin is a disulfide-linked trimer and each monomer unit binds a sugar ligand. A calcium ion is coordinated in the sugar binding site, and the two exocyclic hydroxyl groups, OH5 and OH6, of galactofuranose, are involved in coordination of the calcium ion. This group epitope is also found in α-KO and α-KDO. Thus, the exocyclic 1,2-diol structure defines the binding to intelectin-1, and can be considered the microbe signature for this lectin. Strikingly, intelectin-1 does not bind Neu5Ac (self) or L,D-heptose (non-self), which also share a 1,2-diol structure in the exocyclic part (McMahon et al., 2020). Neu5Ac is frequently found in the non-reducing terminal of mammalian glycans on proteins and lipids while heptose constitutes the partially conserved lipopolysaccharide (LPS) inner core of Gram-negative bacteria. A docking study of Neu5Ac with intelectin-1 suggests charge repulsion and steric interaction problems which inhibit binding. Furthermore, the structures of glycans containing exocyclic vicinal diols were extracted from the PDB, and their favorable conformers were analyzed. The analysis suggests that the exocyclic diol conformation of L,D-heptose is different from that of KDO/KO, and not suitable for binding to intelectin-1 (McMahon et al., 2020). Interestingly, surfactant protein D (SP-D) interacts with heptose (Reinhardt et al., 2016), and X-ray crystallographic analysis shows the interaction of SP-D with the exocyclic diol structure of L,D-heptose (Wang et al., 2008). A detailed analysis of the diol recognition modes by intelectin-1 and SP-D would be interesting.

## Recognition of Oligosaccharide with Unique Linkage and Aglycon

Lectins are typically categorized according to their folds and their monosaccharide specificity. This classification is based on the fact that lectins in a similar fold can recognize a single key monosaccharide residue using the monosaccharide-binding site. Some lectins, however, have an additional sugar binding site, adjacent to the primary binding site. The presence of the secondary binding site allows the lectin to bind certain oligosaccharides with higher specificity. For example, such binding mode is seen in crystal structure of E-selectin (Somers et al., 2000). In the crystal structure of E-selectin lectin domain in complex with an endogenous sialyl-Lewis^X^ [NeuAcα2-3Galβ1-4(Fucα1-3)GlcNAc] (PDB ID: 1G1T), fucose residue interacts with the primary binding site via calcium ion, while galactose and sialic acid residues interact with the positively charged secondary subsite. Even though microbe glycans are composed of common monosaccharides such as glucose or mannose, the linkage can be unique to microbe and be used to recognize the oligosaccharide as a non-self.

For example, β-glucan is composed of glucose residues also found in mammalian N-glycans and glycolipids. However, the Glcβ1-3Glc repeating unit is not found in mammalian glycans. The recognition of β-glucan as non-self must be because of a lectin specific to oligo/polysaccharides with this unique linkage. Dectin-1 is a mammalian β-glucan receptor whose lectin domain is responsible for Ca^2+^-independent β-glucan binding. The minimum length of β-glucan chain required for detectable binding to Dectin-1is a 10 or 11-mer, as determined by a glycan microarray experiment (Palma et al., 2006). Although the atomic details of the dectin-1-β-glucan complex are not known, the crystal structure of ligand-free murine Dectin-1 lectin domain has been reported (Brown et al., 2007). The lectin domain shows a typical C-type lectin fold composed of two anti-parallel β-sheets and two α-helices. An NMR interaction study using a short β-glucan chain, suggests that the center of the short β-glucan chain is indeed recognized by Dectin-1 (Tanaka et al., 2012; Hanashima et al., 2014). The helical nature of the β-glucan chain increases with increasing chain length. Chain-length dependent interaction has been seen, which may reflect a conformational requirement for helix formation to achieve binding to Dectin-1. Furthermore, side chain branching from the β-glucan main chain can affect its affinity toward Dectin-1 (Adams et al., 2008). Further structural analyses are required to understand the binding mechanism.

**FIGURE 3:**
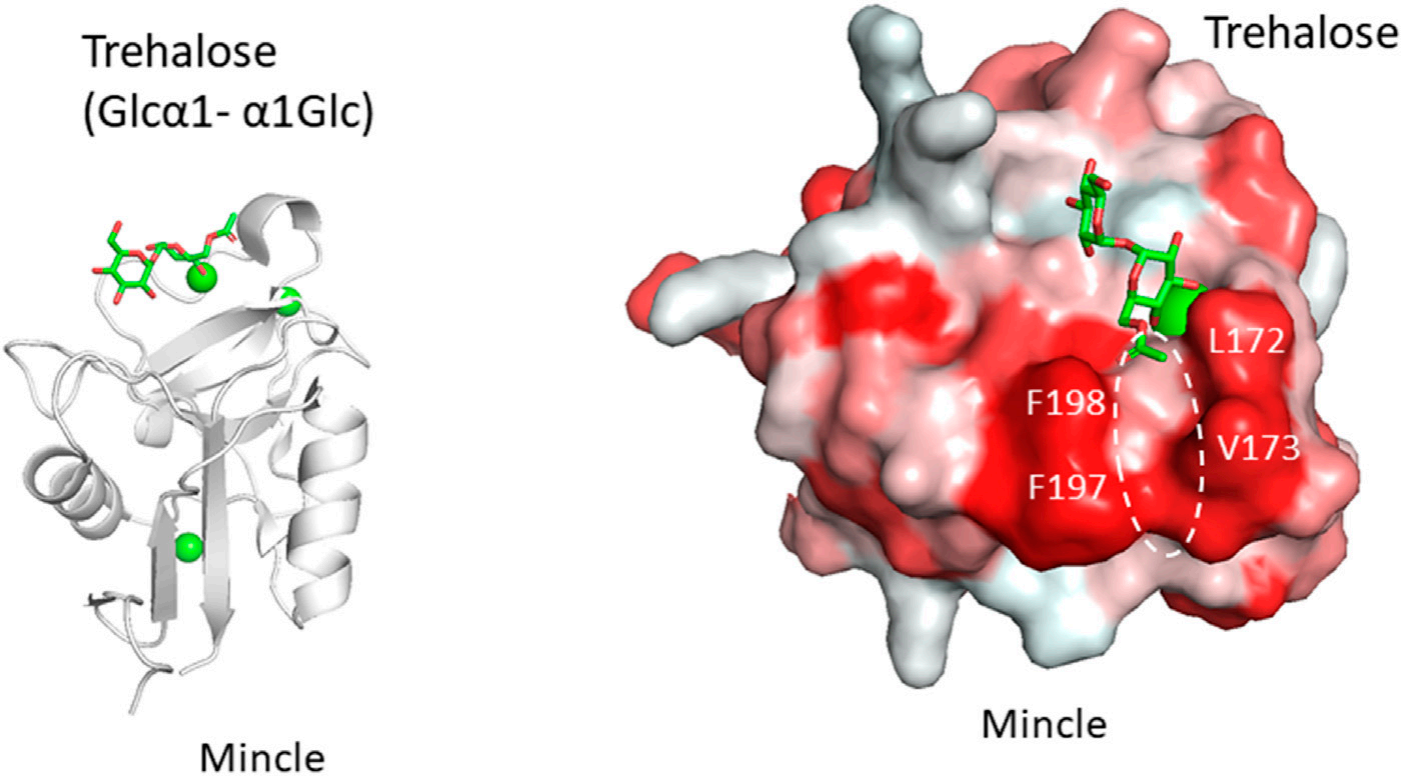
Trehalose (Glcα1-α1Glc) recognition by Mincle (PDB ID: 4ZRV). Overall structure of Mincle lectin domain with trehalose ligand **(A)** and close-up view of ligand-binding site with trehalose monobutylate **(B)**. Calcium ion is shown as a green sphere and the ligand in stick representation. In the right panel, hydrophobic surfaces are colored in red and the putative acyl chain-binding site is indicated with a dotted circle.

Another example is the recognition of the unusual mycobacterial glycolipid with a trehalose structure, Glcα1-1Glcα. This unique α1-α1 linkage is not found in mammals and the disaccharide unit is an important flag for the presence of mycobacteria. Trehalose dimycolate presented on the surface of *Mycobacterium tuberculosis* and the binding of a lectin undoubtedly initiates an immune response. Macrophage-inducible C-type lectin, Mincle is involved in the recognition of this key glycolipid. Mincle is a type II transmembrane lectin receptor and has been reported to bind pathogenic fungi and mycobacteria (Ishikawa et al., 2009; Yamasaki et al., 2009; Ishikawa et al., 2013). Crystal structures of Mincle C-type lectin domain in complex with trehalose and acylated trehalose analogs have been reported (Feinberg et al., 2013; Feinberg et al., 2016) (Figure 3). One glucose residue of trehalose is coordinated with Ca^2+^ as is the case for many other C-type lectins, while the other glucose residue interacts with the secondary binding site, a feature unique to Mincle. This additional binding makes Mincle specific against the trehalose structure. In addition to this secondary site, a hydrophobic shallow groove is found adjacent to the primary binding site. This site may accommodate the acyl chain connected to the six position of the primary glucose residue. Such binding is supported by mutagenesis of amino acid residues in the hydrophobic groove. In a crystal structure, an acylated trehalose analog is bound to Mincle with the acyl chain facing toward the hydrophobic groove (Feinberg et al., 2016). Interestingly, it has been reported that Mincle also binds to other hydrophobic endogenous molecules, namely, cholesterol crystal (Kiyotake et al., 2015), cholesterol sulfate (Kostarnoy et al., 2017), and β-glucosyl ceramide (Nagata et al., 2017). These compounds all have a hydrophobic nature, suggesting the importance of hydrophobic interaction with Mincle. Glycosphingolipid β-glucosyl ceramide is an endogenous lipid found as a metabolic intermediate in human cells. The accumulation of β-glucosyl ceramide in certain diseases, such as Gauchers disease, may activate the immune response through Mincle. The unfortunate recognition of self and subsequent immune activation may occur in certain disease conditions where the amount of self-ligand and/or lectins is abnormally increased. A recent report demonstrated that β-glucosylceramide in combination with free cholesterol binds Mincle as an endogenous ligand to induce cell death during sustained inflammation after acute kidney injury (Tanaka et al., 2020).

Analogous to Mincle, the C-type lectin DCAR (dendritic cell immunoactivating receptor) is a receptor for acylated phosphatidyl-myo-inositol mannosides, which are expressed on the surface of mycobacteria. There is a crystal structure of DCAR lectin domain in complex with acyl chain-free, inositol-monophosphate dimannose at 1.8 Å (Omahdi et al., 2020). The 3D structure shows that a mannose residue interacts with a calcium ion in the primary sugar-binding site in the canonical way. Importantly, a hydrophobic groove is found alongside which likely accommodates the acyl chains. Mutagenesis of the hydrophobic groove supports this idea. The simultaneous recognition of the acyl chains (aglycon part) and the sugar moiety will increase the specificity to bacterial ligands.

ZG16p is a mammalian Jacalin-related mannose-binding lectin with a β-prism fold which also binds to phosphatidyl-*myo*-inositol mannosides (Kanagawa et al., 2011; Kanagawa et al., 2014; Hanashima et al., 2015). Solution NMR and docking suggest the mannose is accommodated in the primary sugar binding site of ZG16p with an additional interaction between inositol phosphate and a Ser side chain. The inositol-mannose linkage and the presence of the phosphate group seem to define the high affinity of ZG16p for phosphatidyl-myo-inositol mannosides. ZG16p also binds to peptidoglycan and Gram-positive bacteria, and in silico docking suggests the binding of peptidoglycan is through both glycan and amino acid moieties (Bergstrom et al., 2016). Further structure-function analyses will extend our knowledge of this small lectin.

### Recognition of Specially Arranged Mannose Residues

**FIGURE 4:**
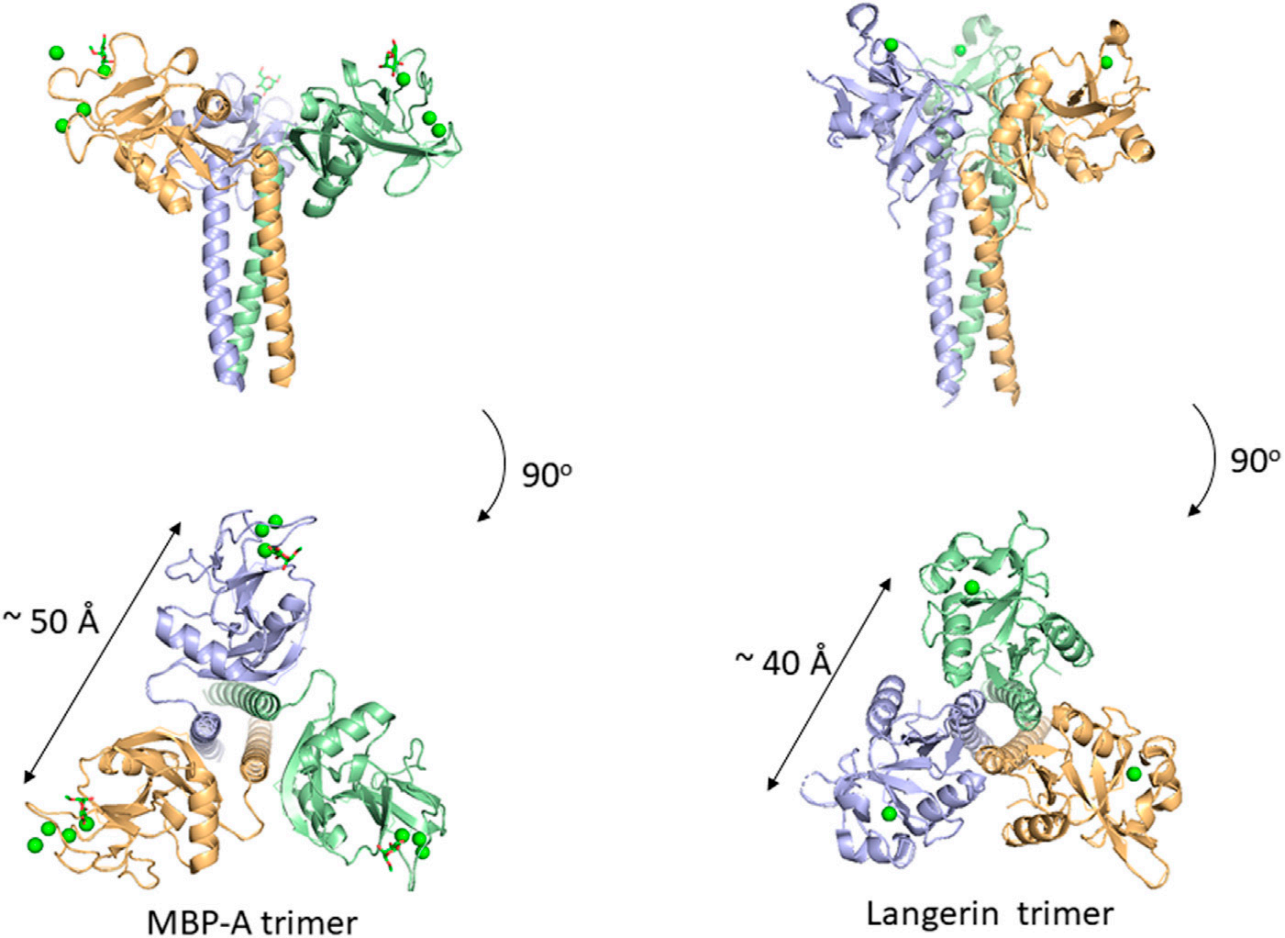
Oligomeric structure of lectin domains guided by the coiled coil region. Trimeric structures of MBP-A (**(A)**, PDB ID: 1KWU) and langerin (**(B)**, PDB ID: 3KQG) are shown. Ligand (O-methyl-mannose) are shown in stick representation for MBP-A. Calcium ions are shown as green spheres.

Many C-type lectins show selectivity toward mannose residues and this mannose binding is utilized for microbe sensing. One of the most studied examples is mannose-binding protein, MBP, which is known to activate the complement lectin pathway. MBP belongs to a structurally homologous family of innate immune defense proteins known as collectins including surfactant protein A (SP-A) and SP-D. Collectin is named after their collagen-like lectin domains. Why does mannose recognition play this role in detecting non-self when mannose residues occur frequently in mammalian N-glycans, e.g., high-mannose glycan. The likely explanation is in the higher spatial density of mannose residues on bacteria relative to that of mammalian glycans. Couple this with the fact that mannose binding lectins are oligomers, a mechanism for enhancing the apparent affinity (avidity) presents itself. Although mannose residues are found in endogenous glycans, the low density and spatial patterns may not fit the binding sites of oligomeric lectins. There are crystal structures of a trimeric MBP-A lectin domain with an α-helical coiled coil region containing various ligands (Ng et al., 2002) (Figure 4). The C-type lectin domain has a Ca^2+^ ion coordinated with the OH3 and OH4 of the mannose. The affinity of 1:1 binding is weak with a dissociation constant of roughly 1 mM, which does not seem enough to trigger an immune response. However, the presentation of multiple binding sites in an oligomer of domains could interact with the multiple terminal mannose residues presented on microbes. The apparent dissociation constant will be roughly equal to the product of each binding constant. The spacing between the sugar-binding sites is around 50 Å, and is eminently suitable for binding packed terminal mannose residues with high affinity, but not single endogenous high-mannose glycans. MBP is in fact found as higher order oligomers organized into bouquet structures of the trimer units (Wallis and Drickamer, 1997).

In addition to MBP, many other C-type lectins form oligomers possibly for the same purpose, namely, detecting multiply-presented mannose residues. Langerin is a C-type lectin with a coiled-coil region and a neck region in a trimeric structure, and has a distance of roughly 40 Å between binding sites (Feinberg et al., 2010). This trimeric structure also seems suitable for detecting pathogens with high mannose density. The paper of Feinberg et al. mentions other examples of oligomeric lectins. A trimeric oligosaccharide ligand with appropriate linker length for the 40 Å distance between each binding site has been reported for Langerin, with 1,000-fold higher affinity over the monomeric ligand (Ota et al., 2018).

### Recognition of Internal Mannose Residues in Mannan

**FIGURE 5:**
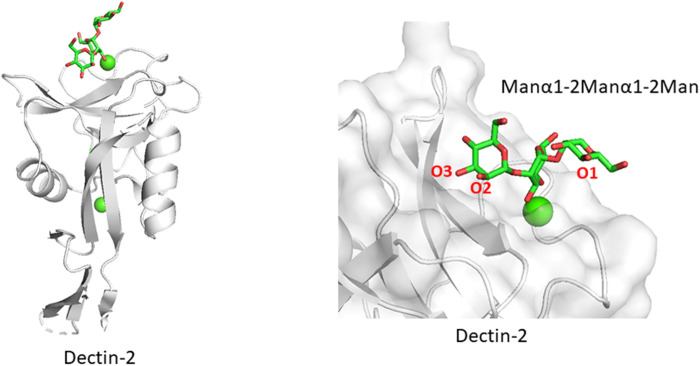
Mannan polysaccharide recognition by Dectin-2 (PDB ID: 5VYB). Overall structure of Dectin-2 lectin domain **(A)** and close-up view of ligand-binding site **(B)** are shown. Manα1-2Manα1-2Man part of the whole N-glycan ligand (Man_9_GlcNAc_2_) is shown in stick representation.

Related to the above section, certain lectin receptors bind mannan and bacterial polysaccharides. Dectin-2 is a C-type lectin expressed on macrophages and involved in the innate immune system. Dectin-2 binds to glycans containing the Manα1-2Man epitope, which is found in fungal mannans and bacterial polysaccharides but also occurs in endogenous high mannose N-glycan. How does Dectin-2 discriminate between the di-mannose structure of self and non-self? The crystal structure of Dectin-2 in complex with an oligosaccharide ligand shows that the binding site of Dectin-2 accommodates internally positioned Manα1-2Man of mannans and other polysaccharides (Feinberg et al., 2017) (Figure 5), whereas other C-type lectins like DC-SIGN and langerin bind only terminal Manα1-2Man structures. Recognition of internal mannose residue is advantageous in that multiple binding sites are presented toward lectin receptor. Dectin-2 is thus suitable for binding to longer mannan polysaccharides. This binding mode is seen in other lectin-polysaccharide and antibody-polysaccharide interaction systems (Nagae et al., 2013; Nagae and Yamaguchi, 2014).

## Future Perspective

Mammalian lectin receptors adopt several strategies for detecting exogenous microbe glycans while tolerating endogenous glycans. Each lectin receptor prefers certain ligands and has been refined to bind to these and only these. Self/non-self-discrimination seems finely balanced, and this balance may be broken under certain disease conditions. Further study of the structural basis of glycan recognition will help gain an understanding of the maintenance of homeostasis, as well as the recognition of damaged cells, and the etiology and occurrence of autoimmune diseases. Structural knowledge is needed for the development of vaccines/drugs against infectious diseases. We know very little about the chemical structures of glycans presented on the surface of the different pathogens. For example, the lipopolysaccharide (LPS) structure is highly diverse, and probably unique for each bacterial strain (Silipo and Molinaro, 2010; Kanie et al., 2019; Shimoyama et al., 2021). An interdisciplinary collaboration, incorporating teams for the determination of microbe glycan chemistry and structure, those contributing glycan microarray analyses, and others focused on 3D structural and database analyses, is needed for a more comprehensive understanding of microbe recognition by lectin receptors.
